# Case report: First experience with stimulating anterior thalamic nuclei in pharmacoresistant epilepsy in Kazakhstan

**DOI:** 10.3389/fnhum.2024.1417382

**Published:** 2024-07-10

**Authors:** Veronika Abzalova, Sholpan Kauynbekova, Gabit Makhambaev, Alexander Dmitriev, Berik Tuleubaev

**Affiliations:** ^1^Department of Surgical Diseases, Medical University of Karaganda, Kazakhstan; ^2^Multidisciplinary Hospital named after Professor Kh.Zh. Makazhanov, Karaganda, Kazakhstan; ^3^Center for New Medical Technologies, Novosibirsk, Russia

**Keywords:** case report, seizures, neuronavigation, neuromodulation, focal

## Abstract

**Introduction:**

Pharmacoresistant epilepsy is a multicomponent disease that can be successfully treated surgically if the surgical tactics are properly defined. We present the first case of stimulation of anterior thalamic nuclei in pharmacoresistant epilepsy in Kazakhstan. This will be a new opportunity for Kazakhstanis diagnosed with epilepsy to achieve stable epilepsy remission.

**Materials:**

The patient was born in 2000. The first episode of tonic clonic seizures with loss of consciousness occurred in 2014. Repeatedly underwent therapeutic and diagnostic measures in the neurological department. The frequency of seizures increased in dynamics. The results of instrumental examination revealed the following morphological changes: Morphological changes: Focal cortical dysplasia (FCD) in the left cingulate gyrus, hypometabolism in the left thalamus and forehead, signs of hippocampal sclerosis on both sides. Electroencephalogram (EEG) shows activity in frontal areas on both sides, more on the right. Based on clinical and instrumental data according to the 2017 ILAE classification, the diagnosis was Structural focal frontal lobe epilepsy with bilateral tonic-clonic seizures. FCD of the left cingulate gyrus. Resistance to antiepileptic therapy.

**Methods:**

The patient was hospitalized in the department of neurosurgery. In light of the evidence indicating structural changes in the brain substance and ambiguous EEG findings, the indications for deep brain stimulation (DBS) of the anterior nucleus (ANT) were made. Electrode implantation was performed under general anesthesia, and preoperative computer tomography (CT) scans were performed using the CRW^®^ stereotactic system in combination with magnetic resonance imaging (MRI) scans using Brainlab Neuronavigation with 3D Atlas to identify the anterior thalamic nuclei.

**Conclusion:**

The observed structural changes in the brain substance and the ambiguous EEG results call into question the efficacy of surgical procedures aimed at removing existing foci or destroying them. Based on the above, as well as the experience of foreign colleagues, the choice of neurosurgeons was DBS ANT. Although the selection of ideal candidates for thalamic stimulation is still controversial, in the described case we were able to achieve control of seizure activity. The patient was seizure free for 2 months after surgery. The patient was discharged on postoperative day 7.

## Introduction

Epilepsy is a common neurological disorder in which abnormal electrical activity in brain cells causes seizures and unusual behaviors and sensations. This limits a person’s daily activities in many ways. It is important to realize that people diagnosed with epilepsy unfortunately face discrimination in society.

This is a heavy burden of society, the global scale, the incidence of which in the world is estimated at 68 cases per 100,000 people per year, which corresponds to 50 million of the world’s population (World Health Organization, 2024). According to the Ministry of Health of Kazakhstan, as of 1 January 2022, 76,678 people with epilepsy were registered in the Electronic Register of Dispensary Patients in Kazakhstan, of whom 27,545 (35.9%) were children under the age of 18 (Ministry of Healthcare of the Republic of Kazakhstan, 2022).

Statistically, approximately 70% of people with epilepsy have their seizures under control, and 30% have drug-resistant epilepsy (Fattorusso et al., 2021).

The International Antiepileptic League defines treatment-resistant epilepsy as the inability to achieve sustained seizure control after two properly selected, scheduled, and tolerated anticonvulsant medications, used as monotherapy or in combination (Patrick et al., 2010). Attempts to reduce seizures with a combination of antiepileptic drugs often result in side effects.

The only solution currently available for this group of patients is surgical treatment. When previous surgery is ineffective or surgical intervention is impossible, neuromodulation is a possible treatment alternative (Herrera et al., 2021). The method does not involve structural, morphologic changes and is aimed at suppressing the activity of the seizure onset zone, which prevents further spread of seizure activity.

Patients with drug-resistant epilepsy are a heterogeneous group with regard to etiology factor, epileptic burden, and drug treatment. This makes it challenging to identify the most suitable candidates for specific surgical procedures. Randomized controlled SANTE studies have demonstrated the efficacy of stimulation in focal temporal seizures. However, the sample size was insufficient to prove the efficacy of the procedure in epileptogenic foci located in other brain areas (Fisher et al., 2010; Salanova et al., 2015). A long-term uncontrolled SANTE study has demonstrated efficacy in the treatment of frontal lobe seizures (Salanova et al., 2021). The Medtronic Registry for Epilepsy (MORE) is an open observational international study that collected prospective and retrospective data to evaluate the long-term efficacy, safety, and effectiveness of the ANT DBS neuromodulation system for the treatment of epilepsy. The majority of patients (96%) were diagnosed with focal epilepsy. The median monthly seizure frequency decreased by 33.1% after two years of treatment (Peltola et al., 2023). In their work, Sitnikov et al. (2017) presented a group of 12 patients who underwent stimulation of the anterior thalamic nuclei. The median seizure frequency reduction after one year was 80.3% (Sitnikov et al., 2017). One patient exhibited idiopathic generalized epilepsy, three exhibited focal epilepsy, and eight exhibited polyfocal epilepsy. The studies described demonstrate a consistent and progressive decline in seizure frequency over time.

The first anterior thalamic nuclei stimulator in the Karaganda region was installed in February 2024. This was a new opportunity for Kazakhstanis to achieve seizure control.

## Patient information

The patient was born in 2000. He had been ill since 2014, when he had his first episode of tonic-clonic seizures with loss of consciousness. The relatives called an ambulance and the patient was admitted to the neurology department. Magnetic resonance imaging of the brain with contrast showed no pathology. EEG did not show any specific epiactivity. There was no family history of epilepsy. Presumptive diagnosis: Unspecified epilepsy with developmental delay, with generalized tonic seizures. Seizures occur once a week. Topamax 100 mg * 2 p/d, Epix 1,000 mg 2 times a day were prescribed. The frequency of seizures tends to increase, the dose of anticonvulsants was increased: Topamax 150 mg * 2 p/d, Epix 1,500 mg 2 times a day.

## Clinical findings

Clinical data: seizures with loss of consciousness during waking and sleeping equally, 3–4 times a week, lasting about 2–3 min. Provocative factors: hot weather, smell of cigarettes, in the company of smokers, the patient does not smoke; thunder, lightning, loud sounds; Types of seizures: focal motor, non-motor-movement stops–the patient can just look and not make any other movements (Epilepsy Foundation Eastern Pennsylvania, 2023)., bilateral tonic-clonic; Postictal symptoms: disorientation, general weakness; Medication history: Levetiracetam- Epix −1,000 mg 2 times a day, 1,500 mg 2 times a day. Topiramate- Topamax- 100 mg * 2 times a day, 250 mg * 2 times a day. Carbamazepine- Carbamazepine- 200 mg twice a day. Valproic acid- Depakine-450 mg * 2 times a day. Lamotrigine- Lamictal- 200 mg * once a day.


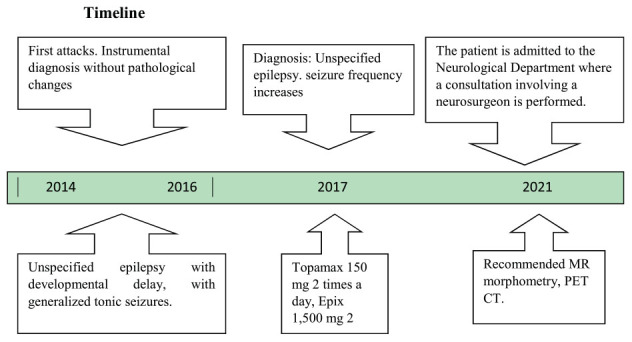


## Diagnostic assessment

**FIGURE 1:**
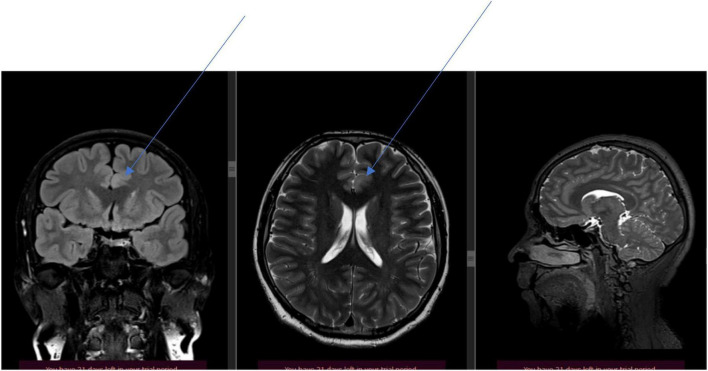
Magnetic resonance morphometry of brain volume: in the left cingulate gyrus, focal cortical thickening (indicated by an arrow) is observed, accompanied by the presence of a thin linear band oriented toward the ipsilateral anterior horn of the lateral ventricle. The volume of focal cortical dysplasia (FCD) was 3.5 cm^3^. The lesion was classified as type 2 FCD in the left cingulate gyrus.

In accordance with the recommendations of the physician, the patient underwent an additional examination in 2021, which included magnetic resonance morphometry of the brain volume (Figure 1). In the left cingulate gyrus, focal cortical thickening (indicated by an arrow) is observed, accompanied by the presence of a thin linear band oriented toward the ipsilateral anterior horn of the lateral ventricle. The volume of focal cortical dysplasia (FCD) was 3.5 cm^3^. The lesion was classified as type 2 FCD in the left cingulate gyrus.

The patient was consulted by an epileptologist. EEG demonstrated the presence of epileptiform activity in the form of spike waves in the frontal areas, with a greater prevalence on the right. Diagnosis: The patient exhibited structural focal epilepsy of the frontal lobe on the left, manifesting as focal motor and non-motor seizures. A positron emission tomography (PET) scan was recommended.

**FIGURE 2:**
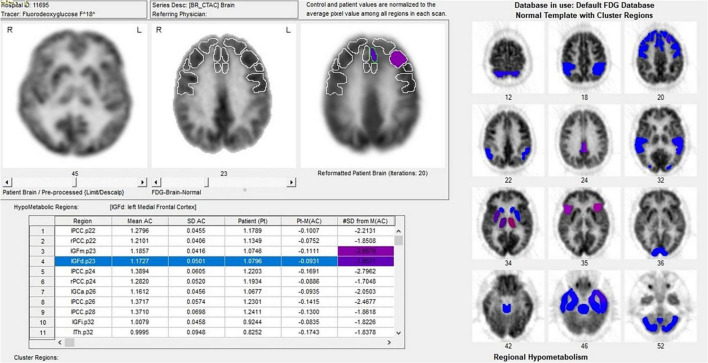
Positron emission tomography: evidence of glucose hypometabolism in the left thalamus, right thalamus, and left superior frontal cortex.

A PET-CT scan was performed in 2022. Conclusion: (Figure 2). Evidence of glucose hypometabolism in the left thalamus, right thalamus and left superior frontal cortex.

In 2023, the frequency of seizures with and without loss of consciousness increased to four to five times a day, each episode lasting approximately ten minutes. The patient was readmitted to the neurology department. A repeat EEG with video monitoring was performed. The epileptiform activity manifested as spike waves in the frontal areas, with a greater prevalence on the right side.

Video monitoring of daily electroencephalogram with seizure recording from 2023: Epiactivity in sagittal, central, frontal, in some places in posterior temporo-occipital leads predominantly on the right. Ictal EEG: abrupt movements of the bent leg, right hand clenched into a fist behind the back, left hand in front, head periodically turns to the left and right, eyes not fixed, blinks periodically.

Brain MRI from 2023: MR signs of hippocampal asymmetry with signs of sclerosis.

As a consequence, morphologic changes: FCD in the left cingulate gyrus, hypometabolism in the left thalamus and forehead, signs of hippocampal sclerosis on both sides. On EEG, activity is in frontal regions on both sides, more on the right.

In light of the aforementioned considerations, surgical interventions involving resection, destruction of foci of epileptiform activity, or hemispheric separation were deemed inadvisable. The patient was therefore indicated for DBS of the ANT.

The patient was admitted to the routine neurosurgical unit with the following diagnosis: Structural, focal frontal lobe epilepsy with bilateral tonic-clonic seizures (according to ILAE classification 2017). Focally cortical dysplasia of the cingulate gyrus on the left. Antiepileptic therapy: Epix 3,000 mg/day, Topamax 500 mg/day. Two days in the hospital before surgery, seizures daily 2 days a day.

## Therapeutic intervention

Preoperative preparation included video-EEG monitoring, 3.0 T optimal MRI, MR morphometry, PET-CT, and a complete neuropsychological evaluation.

Electrode implantation was performed under general anesthesia using the CRW^®^ Stereotactic System, and a preoperative CT scan combined with MRI was obtained on the Brainlab Neuronavigation with 3D Atlas, allowing identification of the anterior thalamic nuclei. T1/T2-weighted 3D, T2-weighted FLAIR images were acquired as part of the imaging protocol for targetics. We targeted the ANT using the Brainlab 3D atlas navigation system and subsequently on sagittal MRI images as a protrusion in the inferior wall of the lateral ventricle.

To avoid postoperative complications, 4 anatomical structures must be identified:

• ANT
• Mammillothalamic tract (MTT)
• Internal/external medullary lamina of thalamus (IML/EML)
• Surrounding veins

The MTT was identified, and the position of the electrode from the inferolateral part of the ATN was planned in order to stimulate the MTT-ATN junction.

We used a transventricular lead trajectory (model 3389 Medtronic Inc.) and tried to approximate the recommended coordinates *X* = 5–6 mm lateral. *Y* = 0–2 mm anterior. *Z* = 10–12 mm superior, based on surgical review: medtronic dbs for epilepsy (Latam Medtronic Academy, 2023).

After determining the coordinates and transferring them to the stereotactic frame attached to the patient’s head, the locations of the trephination holes outside the projection of the major vessels, sulci, and gyrus were determined. The trephination hole was overlaid, TMO coagulation was performed, which was opened transversely. A guide cannula was inserted under direct X-ray control and advanced deep into the brain to a point 10 mm from the desired target, then the electrode was inserted and the cannula withdrawn under dynamic direct X-ray control.

The procedure was repeated on the contralateral side. Then we proceeded to the generator immersion phase, for which a pocket was surgically created in the subclavian region and the generator was hidden under the fascia of the pectoralis major muscle. Stimulation electrodes were connected to the generator via extensions under the skin of the neck and head. After the system was assembled, a test stimulation was performed using the programmer. The system performed satisfactorily. Stimulation parameters were set intraoperatively:

– Stimulation frequency is 140 Hz.
– Pulse width–90 microseconds.
– Cyclicity −1 min of work, 5 min pause.
– Amplitude −5 volts.

**FIGURE 3:**
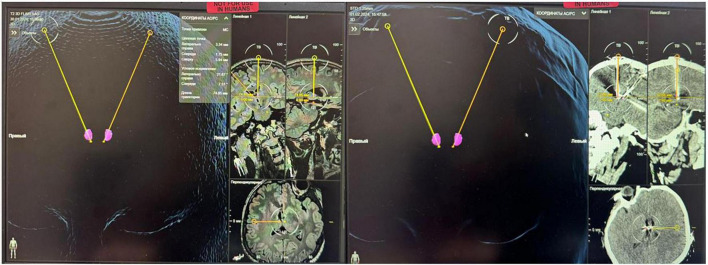
The electrode placement site was confirmed by postoperative CT scan of the alignments on Brainlab with preoperative planning.

The electrode placement site (Figure 3) was confirmed by postoperative CT scan of the alignments on Brainlab with preoperative planning.

## Follow-up and outcomes

In the 3 weeks before hospitalization, seizures with a frequency of 2–3 times a day in the patient’s electronic health passport, ambulance calls daily, in order to control the seizure. During the two days spent in the hospital before surgery, seizures were daily 2 days a day. No seizures have been recorded since the operation during 2 months of follow-up. The patient was discharged from the hospital on the 7th day after the operation. Antiepileptic therapy: Epix 3,000 mg/day, Topamax 500 mg/day.

## Conclusion

The deep and medial location of the cingulate gyrus, the diversity of symptoms and seizures, and the scalp electroencephalogram data together created difficulties in diagnosis and choice of surgical tactics. Presence of changes on PET CT data indicating extensive areas of glucose hypometabolism, which is probably related to seizure pathways (Kumar and Harry, 2017). Visualization of hippocampal asymmetry on MRI. All of the above structural changes were regarded by the neurosurgeon as secondary epileptic foci. Secondary epileptic foci develop over time. Initially, these secondary epileptic foci may be dependent on the primary focus, in which case they tend to disappear after the removal of the primary focus. Over time, these secondary epileptic foci may become independent and are a significant contributor to surgical failure in patients with long-standing intractable epilepsy. In light of the aforementioned considerations, neuromodulation of the anterior thalamic nuclei was deemed the most optimal approach in the described case (Kumar and Chugani, 2009).

## Discussion

Seizures originating in the cingulate gyrus are challenging to localize and characterize using scalp EEG due to their deep location, tendency to spread contralaterally, and secondary bilateral synchrony on EEG (Kerezoudis et al., 2022). One limitation of this study was the lack of availability of stereo EEG. We used all available diagnostic methods in Kazakhstan to verify the diagnosis. In the described case, we expected the stereo EEG to confirm our judgments about seizure initiation and propagation. The judgment was based on the results of instrumental studies, particularly PET CT, which suggests the pathways of seizure activity. EEG traditionally tries to determine the area of seizure initiation, but stereo EEG can add information about more distant structures involved in amplification. The cingulate gyrus is the principal component of the limbic system and is connected to all other structures within the system via neural pathways. The seizure activity of the cingulate gyrus extends to the thalamic nuclei (Chang and Shyu, 2014). The anterior thalamic nuclei are connected to the hippocampus via the mammillothalamic tract and vault, and then project to the cingulate gyrus and neocortex (Jankowski et al., 2013; Weininger et al., 2019). This also supports the propagation of seizure activity between the primary focus and subcortical regions. Consequently, these nuclei may play a pivotal role in the remote control of seizure activity and represent an intriguing target for DBS.

Each identified case of drug-resistant epilepsy has individual etiologic features, seizure frequency, and drug history. For the prediction of results, we have provisionally relied on efficacy data from the internationally renowned SANTE and MORE studies. These studies emphasized that the efficacy of therapy depended on the region of seizure occurrence. The median reduction in seizure frequency in the temporal lobe was 44% after 1 year and 76% after 5 years. While for frontal seizures it was 53 % after 1 year and 59 % after 5 years (Salanova et al., 2015). The median change in temporal seizures ranged from −27.9% after 1 year to 32.6% after 2 years of follow-up; frontal seizures also decreased (−18.4% to −34.7%) (Peltola et al., 2023). And also according to the results of our closest colleagues, the authors note the usefulness of the method in suppressing secondary generalized seizures, while simple partial seizures are less amenable to treatment (Sitnikov et al., 2017). Which, according to the ILAE 2017 classification, fits the description of seizures in the described case (Epilepsy Foundation, 2016).

The results of PET-CT, MRI and the equivocal conclusion of scalp EEG did provide insight into the pathways of seizure activity, indicating that ANT DBS was the most appropriate solution in the described case. The patient is under observation and will continue to be monitored. Two months have passed since surgery and no seizures have occurred. In the future, an EEG is planned, followed by a consultation with an epileptologist to decide whether it is necessary to change the dosage of antiepileptic drugs.

Today, the scope of DBS has expanded to multiple brain diseases with the goal of modulating neural circuits. The DBS Think Tank estimates that 208,000 deep brain stimulation (DBS) devices have been implanted worldwide to treat a range of neurological and neuropsychiatric disorders (Vinata and Karl, 2021). The DBS Think Tank East, in its study, addresses the principal challenges to DBS accessibility in Asia and Oceania. The most active countries, both practically and theoretically, are Japan, Korea, Israel, Australia and New Zealand. The scientific research on DBS is not yet widely conducted in Asia. The primary obstacle to research activities is the dearth of financial support from governmental and industrial sources. Approximately 77% (38) of Asian countries have no DBS publications. In comparison, 402 DBS publications were found from Canada alone (Zhang et al., 2020).

Despite the advancement of DBS in Asia, numerous obstacles and challenges remain. These include limited financial resources, a lack of specialized staff, and the absence of a distinct field of functional neurosurgery. A significant number of countries continue to lack access to the DBS. Most of the available publications are from Turkey, Israel and Iran. This situation is a cause for concern, particularly given the high prevalence of movement disorders and other conditions treatable with DBS. According to the World Health Organization (WHO), in high-income countries, approximately 49 individuals per 100,000 people are diagnosed with epilepsy each year. In low- and middle-income countries, the incidence of epilepsy can be as high as 139 per 100,000 individuals (World Health Organization, 2024).

Thanks to the combined efforts of neurosurgeons, neurologists, and epileptologists, Kazakhstanis diagnosed with pharmacoresistant epilepsy have access to a wide range of surgical methods of treatment, including DBS ANT. Our clinic offers a state-of-the-art EEG technology, stereo EEG, since April 2024, which provides a more accurate diagnosis for patients with controversial scalp EEG readings.

## Data availability statement

The datasets presented in this article are not readily available because of ethical and privacy restrictions. Requests to access the datasets should be directed to the corresponding author.

## Ethics statement

The studies involving humans were approved by the Local Bioethics Commission of the Medical University of Karaganda. The studies were conducted in accordance with the local legislation and institutional requirements. Written informed consent for participation in this study was provided by the participants’ legal guardians/next of kin. Written informed consent was obtained from the individual(s) for the publication of any potentially identifiable images or data included in this article.

## Authors contributions

VA: Writing – original draft, Writing – review & editing, SK: Conceptualization, Methodology, Writing – review & editing. GM: Writing – review & editing. AD: Writing – review & editing. BT: Conceptualization, Methodology, Writing – review & editing.
